# Posterior tibial nerve stimulation for overactive bladder—techniques and efficacy

**DOI:** 10.1007/s00192-019-04186-3

**Published:** 2019-12-18

**Authors:** Alka A. Bhide, Visha Tailor, Ruwan Fernando, Vik. Khullar, Giuseppe Alessandro Digesu

**Affiliations:** St Mary’s Hospital, Imperial College NHS Trust, London, UK

**Keywords:** Tibial nerve stimulation, Percutaneous, Transcutaneous, Implantable, Overactive bladder, Neuromodulation

## Abstract

The ideal treatment for overactive bladder is still elusive. In those where medication fails to improve symptoms options include invasive treatments such as botulinum toxin-A, sacral neural stimulation or posterior tibial nerve stimulation. Scientific professional society guidelines advise percutaneous posterior tibial nerve stimulation as a third line treatment option only after multi-disciplinary team review as well as failure of both conservative and pharmacological management. The aim of this article is to review all techniques for tibial nerve stimulation and their efficacy.

## Introduction

Overactive bladder (OAB) is defined by the International Continence Society as ‘urinary urgency, with or without frequency and nocturia, with or without urgency urinary incontinence, in the absence of urinary tract infection or other obvious pathology’ [1]. Treatment recommendations from various scientific bodies include behavioural therapy and oral medications with antimuscarinics, beta 3 adrenoreceptors or intravesical botulinum toxin A injections [2–4]. In patients who do not respond to these therapies the use of tibial nerve stimulation has been recommended with various grades of evidence [5].

Tibial nerve stimulation (TNS) is a form of neuromodulation involving the use of electrical impulses to address urinary symptoms. The aim of neuromodulation is to target the innervation system of the lower urinary tract. The posterior tibial nerve is a distal branch of the sciatic nerve that originates in the pelvis (L5–S3 spinal roots) and descends towards the lower extremities. Stimulation of the posterior tibial nerve delivers retrograde neuromodulation to the sacral nerve plexus that controls the bladder function. Stimulation can be achieved via a percutaneous needle electrode; a transcutaneous surface electrode or more recently wireless implantable tibial nerve stimulators are being trialled and developed. We describe these techniques further:

### Percutaneous tibial nerve stimulation (PTNS) [6]

The posterior tibial nerve is stimulated by inserting a 34-gauge needle 4–5 cephalad to the medial malleolus. Once the current is applied, the flexion of the big toe or the movement of the other toes confirms the correct positioning of the needle electrode. The electrical current is a continuous square wave form with a duration of 200 Us and a frequency of 200 Hz. The intensity of the current is determined by the highest level tolerated by the patient. The stimulation sessions last for 30 min.

### Transcutaneous tibial nerve stimulation (TTNS) [7]

Posterior tibial nerve stimulation is given via two 50 mm × 50 mm electrode pads. The live pad is placed posterior and superior to the medial malleolus and the ground pad is placed approximately 10 cm cephalad to this. Continuous stimulation at a pulse width of 200 ls and a frequency of 10 Hz is used. The amplitude was set to produce a sensory stimulus in the ipsilateral foot, at an intensity tolerable to the patient. Stimulation is given for 30 min.

### Implantable devices

Implantable devices to stimulate the tibial nerve were first described by Van der Pal et al. (2006) [8]. They published outcomes using a subcutaneous implant Urgent-SQ (Uroplasty, Inc., Minnetonka, MN, USA) in eight patients. At 9 years seven patients had the device in situ with three patients continuing to use it [9].

A newer battery-less RENOVA iStim™ implant (BlueWind Medical, Herzliya, Israel) [10] is wirelessly powered by an external control unit (ECU), which provides the therapeutic parameters. Tibial nerve stimulation is only delivered when the ECU is worn. The implant consists of a 25-mm cylinder with a diameter of 3.4 mm and four small fixating wings. The cylinder consists of an electrical power receiver and two bipolar electrodes. Stimulation can be performed with a pulse width of between 50 and 800 µs, a frequency of 5, 10, 20 and 40 Hz and an amplitude in the range of 0 to 9 mA. Patients can adjust only the amplitude between a patient-specific set minimum and maximum.

This implant is placed over the tibial nerve in an open surgical procedure with antibiotic prophylactic cover usually with local anaesthetic. A 5-cm incision is made 3 cm superior and 2 cm posterior to the medial malleolus. The tibial bundle is identified including the tibial nerve and the electrode is placed near the nerve and secured with non-absorbable sutures to the fascia. A test stimulation with observation of a motor response is performed to check proper functioning and positioning of the electrode. A pressure bandage is applied for 24 h and patients are instructed to slowly increase walking activities after 24 h. One month after insertion the operation system is activated using the standard starting parameters of a pulse width of 200 µs and a stimulation frequency of 20 Hz. The minimum amplitude is set to the amplitude with which the patient experiences the first sensation of stimulation and the maximum amplitude was set at the highest tolerable level. Patients are asked to wear the rechargeable ECU six times per week for 30 min at a comfortable amplitude to provide treatment stimulation. After activation the parameters can be adjusted if necessary.

In development and currently undergoing feasibility trials is the battery-powered eCoin™ (Valencia Technologies Corp., CA, USA) implant as well as the StimRouter™ (Bioness, CA, USA) implant. Initial results are promising with further studies anticipated [11].

## Summary of the scientific evidence

### PTNS and anti-muscarinic treatment

Four randomized controlled trials (RCT) have been conducted comparing PTNS with antimuscarinic medication in patients with overactive bladder. Two studies described the use of Stoller afferent neurostimulation (SANS-posterior tibial nerve stimulation with a needle) either in isolation or combined with an antimuscarinic. Results differed with one stating no difference in urgency or frequency between those using neurostimulation alone and those using it together with an antimuscarinic [12] and the other finding a significant decrease in OAB symptom severity in the combination group compared to the antimuscarinic group alone [13].

The largest study with 105 women with OAB compared solifenacin alone with PTNS alone with solifenacin and PTNS combined [14]. Results demonstrated that PTNS was more effective than solifenacin, but that combination therapy was the most effective and demonstrated more durability than PTNS and solifenacin alone.

### PTNS with sham-controlled treatment

Two double-blind randomized controlled trials have been carried out comparing PTNS with sham treatment. In the first smaller study 71% of patients in the PTNS group (18 patients) were classed as responders compared with zero in the placebo group (17 patients) [15]. The SUmiT trial in the USA [16] consisted of 220 participants (both male and female) with OAB. This multicentre trial compared the efficacy of PTNS with sham treatment through 12 weeks of therapy with 110 participants in each arm. The global response assessment for overall bladder symptoms showed PTNS led to a significant improvement in bladder symptoms compared with the sham group. Three-day bladder diary parameters showed the PTNS group to be superior to the sham group with greater improvements in frequency, nighttime voids, urgency and urge incontinence (statistically significant).

### PTNS compared with transvaginal stimulations and pelvic floor muscle training

It is thought that pelvic floor muscle stimulation leads to reflex contraction of the striated paraurethral and periurethral muscles with simultaneous reflex inhibition of the detrusor muscle. Ugurlucan et al. (2013) [17] compared the effects of transvaginal electrical stimulation (ES) and PTNS in a randomized controlled trial. The study demonstrated that there was no statistically significant difference between the two groups in objective measurements. Quality of life assessments showed improvements in both groups but only a significant difference in the social limitations domain between ES and PTNS (ES significantly better than PTNS).

Another randomized controlled trial compared PTNS and ES with pelvic floor muscle training (PFMT) in 60 women with OAB with 30 participants allocated to each arm [18]. The PTNS group demonstrated a significant reduction in frequency, nocturia and urge incontinence. When the two groups were compared after treatment, women treated with PTNS showed statistically significant improvement compared with those treated with ES and PFMT. Quality of life assessment comparing post-treatment data also showed patients treated with PTNS had better results than those treated with ES and PFMT.

### Non-comparative studies of PTNS and long-term outcomes

A 6-week prospective observational study [19] assessed the efficacy of a shortened 6-week protocol of PTNS in 43 women with refractory OAB; 68.7% of women were classed as positive responders and quality of life scores improved by 25% in the responders. This trial suggests that a shortened 6-week period of weekly PTNS can be beneficial perhaps making it more appealing to patients and more cost effective. However an earlier study by Van der Paal et al. (2006) [20] assessed whether maintenance PTNS treatment was necessary in 11 patients with refractory OAB. This study, although with small numbers, demonstrated that continuous therapy is necessary in patients with OAB successfully treated with PTNS and the efficacy of PTNS can be reproduced in patients formally treated successfully. The data from these two studies together reveal that although a shortened protocol of PTNS can be effective patients will most likely need maintenance therapy to continue the beneficial effects.

Iyer et al. (2018) [21] retrospectively reviewed 183 patients with refractory OAB over a 9-year period who received 30-min sessions of PTNS for 12 weeks. There was a statistically significant improvement in urinary frequency, nocturia and urge incontinence episodes in the PTNS group, with the effect seen by week 10 of treatment; 61.5% of participants self-reported > 50% improvement in symptoms with the number of PTNS sessions increasing the odds of subjective success. In addition to the number of sessions as a success predictor, a retrospective study by Rostaminia et al. (2018) [22] showed that a history of depression/anxiety and severe baseline urgency urinary incontinence were positive predictors of successful PTNS outcome in women with OAB. Review of urodynamic data in 90 patients with OAB treated with PTNS showed that patients without detrusor overactivity may respond better to PTNS suggesting that urodynamics may help in patient selection [23].

The OrBIT trial (phase 2) assessed long-term use of PTNS in 33 patients with OAB [24]. After 12 weeks of weekly PTNS sessions as part of phase 1, participants were offered an additional 9 months of treatment. Over the 9 months there was an average of 21 days between treatments. Global response assessments showed sustained improvement at 6 and 12 months in 94% and 96% of responders respectively. At 12 months there was a significant improvement in frequency, urge incontinence, nocturia and voided volume compared with baseline. This study further demonstrated PTNS to be a viable option for long-term therapy for OAB.

Peters et al. (2013) [25] also reported the long-term efficacy and safety of PTNS for OAB after 3 years of therapy in 29 patients. These patients then underwent a 14-week tapering protocol followed by a personal treatment plan aimed at sustaining OAB symptom improvement. Overall 77% of patients maintained moderate or marked improvement in OAB symptoms at 3 years with an average of one PTNS treatment a month. This again supports the idea that those who respond initially to PTNS may benefit from top-up treatments to maintain symptom improvement.

A small Spanish study by Arrabal-Polo et al. (2012) [26] recruited 14 women with OAB refractory to anticholinergic treatment. They underwent 14 sessions of PTNS: 8 weekly sessions, followed by 4 sessions every 15 days and then 2 monthly sessions. They showed a significant reduction of frequency, urgency and urge incontinence and 50% of patients felt a subjective improvement in symptoms. Another Spanish group used a similar protocol and extended it for 30 months in 200 women with refractory OAB8 [27]. They underwent weekly PTNS for 8 weeks, then every 2 weeks for 8 weeks, then monthly for 8 weeks for 6 months. The results demonstrated a clinical improvement in 90.5% of patients at the end of treatment. In the 60 patients that had long-term follow-up there was maintenance of improvement in day- and nighttime frequency at 6 months, with satisfactory benefit at 12 and 18 months with no significant worsening. At 24 and 30 months no significant difference was seen in daytime frequency compared with results immediately after treatment. This outcome suggests that retreatment may need to be offered at this time point in patients who have benefited from PTNS in the past.

### PTNS in specific groups

PTNS has also been investigated in specific conditions. Zecca et al. (2014) [28] carried out a non-comparative prospective study on 83 multiple sclerosis patients with refractory OAB symptoms. Participants received PTNS for 30 min once a week for 12 weeks. Sixty-one per cent were classed as responders (> 50% improvement in lower urinary tract symptoms according to the PPBC) after 12 weeks of PTNS.

Long-term PTNS treatment in MS patients with neurogenic OAB symptoms was investigated in a non-comparative prospective study by Kabay et al. (2017) [29]. Twenty-one patients completed 1-year PTNS treatment with a tapering protocol of 14-day intervals for 3 months, 21-day intervals for 3 months and 28-day intervals for 3 months. There was a significant improvement in daytime frequency, nocturia, urgency episodes, voided volumes and urge incontinence episodes at the 6, 9- and 12-month timeline compared with baseline.

A meta-analysis published this year has reviewed the role of PTNS on sexual function in women with pelvic floor dysfunction including overactive bladder [30]. Although the numbers are small there is evidence to suggest that PTNS has a positive effect on sexual function and further research is recommended.

### Transcutaneous tibial nerve stimulation (TTNS)

TTNS is an alternative method of stimulating the posterior tibial nerve using a patch rather than a needle electrode. One study randomized women into three groups; group 1 had TTNS twice a week for 30 min for 12 weeks, group 2 received slow-release oxybutynin 10 once daily for 12 weeks and group 3 received both treatments [31]. All groups showed improvement in OAB symptoms and quality of life scores. However, the combined treatment was more effective than single treatment. In addition, TTNS alone or in association with oxybutynin demonstrated longer lasting results in terms of clinical symptom improvement and QoL.

Another prospective randomized trial assessed TTNS (*n* = 36) versus extended release oxybutynin (ERO) (*n* = 34) in OAB patients [32]. The regime involved TTNS twice a week for 30 min for 12 weeks or 10 mg ERO once daily. There was a statistically significant reduction in frequency, urgency episodes and UI episodes compared with baseline; however there was no significant difference between the two groups overall.

A recent study randomized 40 women with nocturia into two groups of weekly TTNS sessions compared with pelvic floor muscle training and behavioural therapy for a 12-week treatment period [33]. Both treatments resulted in an improvement in the quality of sleep with a reduction in the number of awakenings to urinate (45% in both groups reduced by 1).

TTNS seems to be as good as PTNS in terms of symptom improvement and may be an option for those patients who find needle insertion unacceptable.

### Implantable devices

The RENOVA iStim™ implantable device is one of the newest peripheral neuromodulation modalities developed by BlueWind Medical, Herzliya, Israel. Breda et al. (2017) [34] carried out a non-comparative study in 14 participants with OAB. The implantable device was inserted using the technique described earlier and was used six times a week for 30-min duration. At 3 months, there was a significant decrease in 24-h frequency. There was also a significant increase in mean micturition volume. Thirteen out of the 14 patients experienced a > 50% improvement in the number of severe urinary urgency episodes. Complications included pain and infection at implantation site requiring oral analgesia and oral antibiotics.

Heesakkers et al. (2018) [35] evaluated the same device over a 6-month period in a prospective study in 36 participants with OAB. The device was used six times a week for 30-min duration for 3 months or three times a week for 6 months. At 6 months 71% of participants had significant improvement in daytime frequency, urgency and urgency incontinence. In terms of the two regimes (3 months and 6 months), 86.4% who displayed clinical success (> 50% improvement) after 3 months maintained this at 6 months. Furthermore, 41.7% who did not experience clinical success at 3 months did so at 6 months.

## Conclusion

The studies presented here show the use of TNS in a heterogeneous group of patients with OAB, refractory OAB and neurogenic OAB, both male and female. The main side effect from the percutaneous approach is pain at the needle insertion site. Inflammation and pain at the insertion site of the implantable device resulted in device removal in one patient in the two studies described here.

The use of PTNS in isolation in patients with OAB does seem to provide improvement in symptoms as evidenced in the two RCTs comparing PTNS with sham treatment. However, the evidence from combination studies with PTNS and an anti-muscarinic demonstrates that the two together provide greater symptom improvement.

The non-comparative studies have demonstrated the efficacy of a shortened 6-week protocol of PTNS with nearly 70% of participants classified as responders. Long-term maintenance is also a valid option especially in those who have responded well to the initial weekly treatment to maintain symptom control. There is evidence to suggest that PTNS in MS and Parkinson’s disease patients leads to symptom improvement both for a 12-week course and in those having 3-month maintenance treatments. Various predictors of treatment success have been identified and it has been shown that when PTNS is incorporated into a patient navigation pathway as a third-line treatment patient utilization and retention are increased [36].

TTNS showed similar results to PTNS; when compared with oxybutynin there was no significant difference in outcome; however when used in combination with oxybutynin, results were better than if either was used in isolation. However, in those with nocturia TTNS and pelvic floor muscle training produce similar results. Recent guidance on female urinary incontinence in the UK has recommended that TTNS should not be offered to treat OAB. They have also recommended that PTNS be offered only after multidisciplinary team review, non-surgical management for OAB has failed and the woman has declined BOTOX or sacral nerve stimulation. Overall the evidence shows that PTNS results short- and long-term OAB symptom improvement. It could be argued that side effects with this treatment are more tolerable than those seen with antimuscarinics such as dry mouth and constipation. In addition, there is concern for non-degenerative cognitive impairment in the elderly with chronic anti-cholinergic drug use.

Technology advances may see a rise in peripheral implantable neuromodulation treatments that allow convenient and personalized TNS to be carried out. There is potential to reduce the travel burden to patients attending for PTNS and provide a less invasive single-step implant procedure compared with sacral neuromodulation therapies. However, further research into long-term outcomes and neuromodulation delivery systems is required.
